# Diabetes mellitus and its associated factors among human immunodeficiency virus-infected patients on anti-retroviral therapy in Northeast Ethiopia

**DOI:** 10.1186/s13104-019-4402-1

**Published:** 2019-07-01

**Authors:** Temesgen Fiseha, Alemu Gedefie Belete

**Affiliations:** Department of Clinical Laboratory Science, College of Medicine and Health Sciences, Wollo University, Dessie, Ethiopia

**Keywords:** Diabetes, HIV, Antiretroviral therapy, Ethiopia

## Abstract

**Objective:**

The aim of this study was to determine the prevalence of diabetes mellitus and its associated factors among human immunodeficiency virus-infected patients on anti-retroviral therapy in Northeast Ethiopia.

**Results:**

A facility based cross-sectional study was conducted among 408 HIV-infected adults (≥ 18 years old) attending an ART clinic in Northeast Ethiopia from January to March 30, 2018. The mean (± SD) age of studied patients was 37 ± 10.3 years, and 273 (66.9%) were female. Of the total participants, 36 (8.8%, 95% CI 6.4% to 11.8%) had diabetes and 61 (15.0%, 95% CI 11.5% to 18.6%) had impaired fasting glucose level (111–125 mg/dl). Only fourteen (3.4%) participants knew their diabetes status during data collection. In the multivariate analysis, older age (age > 45 years; AOR = 3.51, 95% CI 1.52–8.10, *P *= 0.003), a family history of diabetes (AOR = 6.46, 95% CI 3.36–21.29, *P *< 0.001), duration of ART (AOR = 2.67, 95% CI 1.16–6.17, *P *= 0.021), and hypertension (AOR = 2.62, 95% CI 1.20–5.72, *P *= 0.016) were independently associated with increased odds of diabetes. These results highlight the need for regular diabetes screening among HIV-infected patients on ART in order to prevent or reduce disease-related outcomes of these patients in this study setting.

**Electronic supplementary material:**

The online version of this article (10.1186/s13104-019-4402-1) contains supplementary material, which is available to authorized users.

## Introduction

Globally, 37 million people are living with the human immunodeficiency virus (HIV). By the end of 2017, 21.7 million people living with HIV were accessing antiretroviral therapy (ART), an increase of 8 million since 2010 [1]. Expansion of access to ART has reduced AIDS-related mortality and increased life expectancy among people living with HIV in both high- and low-income countries [2–4]. With prolonged survival of persons with HIV, chronic comorbidities including diabetes have emerged as significant causes of morbidity and mortality among these patients with access to ART [5, 6]. Diabetes mellitus is now recognized as an important chronic comorbid condition occurring in people living with HIV, and is associated with increased morbidity and mortality [7]. In patients with HIV, diabetes has been shown to be associated with an increased risk for hospitalization, and adverse cardiovascular and renal outcomes, including progression to end-stage renal disease that consequently reduces life expectancy and add to the already high costs of treatment for this population [8–10].

HIV-infected patients on combination ART have a higher prevalence and earlier onset of diabetes when compared with HIV-uninfected individuals [11, 12]. Several risk factors have been shown to contribute to the high prevalence of diabetes in such HIV-infected population, including older age, male sex, family history of diabetes, duration of HIV infection, CD4 count, high body mass index, and hepatitis C virus (HCV) co-infection [7, 12, 13]. In addition, the use of certain medications included in combination ART regimens have been also shown to contribute to the development of diabetes in people living with HIV [14]. In this regard, a recent meta-analysis of data from 41 observational studies reported that the risk of diabetes among ART-exposed patients was as four times higher compared with treatment-naive patients, and have suggested that ART may potentially be the single most consistent determinant of diabetes in people living with HIV [15].

Although HIV-infected patients on ART are at greater risk of developing diabetes and thus, suffer extra morbidity and mortality than those without the disease; relatively little is known about its burden among HIV infected patients in Ethiopia. The aim of this study was to determine the prevalence of diabetes mellitus and its associated factors among HIV-infected patients in Northeast Ethiopia.

## Main texts

### Methods

#### Study design, settings and participants

This cross-sectional study was conducted from January to May 2018 at the ART clinic of Dessie Referral Hospital (DRH), Northeast Ethiopia. DRH is found in Dessie town of Amhara regional state, which is located 401 km northeast of the capital Addis Ababa, Ethiopia. The hospital serves as a referral center for the Wollo and surrounding zones and provides HIV/AIDS interventions including free diagnosis, treatment and monitoring. HIV-infected adults (aged > 18 years) attending the ART outpatient clinic for regular follow up, who were on ART for at least 12 months and willing to provide consent were included in the study. Patients were excluded if they were pregnant (women), hospitalized, with a clinical thyroid disease and/or with critical illness that rendered them unable to answer the questionnaires.

#### Sample size determination

The sample size was calculated by considering 50% prevalence rate of diabetes mellitus. Using single population proportion formula, with the following parameters: Z = the level of statistical significance with a 95% confidence interval (CI) of 1.96, and a precision level of 0.05 then the minimum sample size obtained was 384. By adding 10% to account for non-respondents, a total of 422 HIV infected individuals on ART were consecutively included in the study.

#### Data collection and laboratory measurements

Participants were interviewed for collecting data on socio-demographic, medical and family history, and behavioral factors (smoking and alcohol consumption) by using a pre-tested structured questionnaire. During the interview process, patients were given an ID number and then had their anthropometric data such as weight (kg), height (m), and blood pressure (mmHg) collected. The body mass index (BMI) was calculated as weight (kg) divided by the square of height (meters). Blood pressure (BP) was measured using a manual sphygmomanometer in a sitting position after 5 min at rest and three measurements were averaged to be recorded. Clinical measures including duration of HIV infection, duration on ART, types of ART-regimens were abstracted from patients’ database. Blood samples were collected in the early morning after an overnight fasting. Fasting plasma glucose was measured by enzymatic GOD-PAP method as mg/dl using A25 Biosystems clinical chemistry analyzer (BioSystems S.A., Barcelona, Spain). CD4 cell count was measured using the BD FACSCOUNT system (Becton Dickenson and Company, California, USA).

#### Definitions

Diabetes mellitus was defined as fasting plasma glucose level ≥ 126 mg/dl on two consecutive study visits [16] and/or a previous diagnosis of diabetes. Hypertension was defined as self-reported use of antihypertensive medication and/or systolic BP ≥ 140 mmHg and/or diastolic BP ≥ 90 mmHg on at least two occasions. Participants were categorized as smokers, if they smoke at least one cigarette for the last 12 months and alcohol consumer, if they consume at least twice weekly of any alcoholic drinks. BMI classified as normal/underweight (BMI < 25 kg/m^2^), overweight (BMI 25–29.9 kg/m^2^) and obese (BMI ≥ 30 kg/m^2^).

#### Statistical analysis

Data were entered into “EpiData version 3.1” and exported to SPSS version 20.0 statistical software for analysis. Data were reported using mean ± standard deviation (SD) for continuous variables and proportions for categorical variables. Comparisons between groups were done by Chi square (χ^2^) test or *t* test as appropriate. Variables having *P*-value < 0.25 in the univariate analysis were included in the multivariate model using logistic regression and the corresponding adjusted odds ratios (AOR) and 95% confidence intervals (CI) were used to identify factors independently associated with diabetes. *P* value < 0.05 was used to indicate statistical significance.

#### Ethical consideration

Study protocol was approved by the Institutional Review Board of College of Medicine and Health Sciences of Wollo University. Permission to conduct the study was also obtained from Dessie Referral Hospital. An informed verbal as well as written consent was obtained from all the patients enrolled. Physicians were informed about diabetic patients for proper management.

### Results

#### Demographic and clinical characteristics of the study participants

Of the total 422 HIV infected patients considered eligible, 408 (66.9% females and 33.1% males) were included in the analysis. The participants’ demographic and clinical characteristics are presented on Table 1. The mean age was 37 ± 10.3 years, ranging from 18 to 73 years. One hundred and fifty (36.8%) participants were receiving AZT containing ART regimens (AZT + 3TC + EFV/NVP) (Table 1).

**Table 1:** Demographic and clinical characteristics of study participants (n = 408)

Characteristics	
Age (year), mean ± SD	37 ± 10.3
Age group, n (%)	
18–34	169 (41.4)
35–45	161 (39.5)
> 45	78 (19.1)
Sex, n (%)	
Male	135 (33.1)
Female	273 (66.9)
Residence, n (%)	
Rural	88 (21.6)
Urban	320 (78.4)
Education, n (%)	
< High school	240 (58.8)
≥ High school	168 (41.2)
Duration of infection, n (%)	
≤ 5 years	227 (55.6)
> 5 years	181 (44.4)
Duration on ART, n (%)	
≤ 5 years	145 (35.5)
> 5 years	263 (64.5)
ART regimen, n (%)	
AZT + 3TC + EFV	66 (16.2)
TDF + 3TC + NVP	71 (17.4)
AZT + 3TC + NVP	84 (20.6)
TDF + 3TC + EFV	76 (18.6)
Other	111 (27.2)
Current smoker, n (%)	16 (3.9)
Alcohol consumers, n (%)	62 (15.2)
Family history of DM, n (%)	42 (10.3).
Body mass index (kg/m^2^), mean ± SD	20.40 ± 2.11
Systolic BP (mmHg), mean ± SD	127.2 ± 12.8
Diastolic BP (mmHg), mean ± SD	82.0 ± 6.0
Hypertension, n (%)	121 (29.7)
CD4 count (cell/mm^3^), mean ± SD	343.1 ± 311.6
Fasting plasma glucose (mg/dl), mean ± SD	105.7 ± 39.0

Other regimens: D4T + 3TC + NVP, D4T + 3TC + EFV, TDF + 3TC + ATV/R, ABC + 3TC + ATV/R, ABC + 3TC + EFV, AZT + 3TC + ATV/R, DDI + ABC + ATV/R

#### Prevalence of diabetes and associated factors

A total of 36 (8.8%) patients had diabetes and 61 (15.0%) had a fasting plasma glucose 111–125 mg/dl. Of the patients with diabetes, 14 (3.9%) knew about their diabetes status. Diabetes prevalence increased significantly with age of the patients, 4.7% for age 18–34 years, 6.8% for 35–45 years and 21.8% for > 45 years, and was significantly higher in older patients (aged > 45 years): 21.8% vs. 5.8% (*P *< 0.001). The prevalence was slightly higher in men (11.9%) than women (7.3%; *P *= 0.129). No significant difference was also detected in diabetes prevalence between rural (10.2%) and urban residents (8.4%; *P *= 0.600).

Diabetes prevalence was significantly higher in participants with less than high school education (*P *= 0.039), longer duration since HIV diagnoses (*P *= 0.035), longer duration on ART (*P *= 0.002), a family history of diabetes (*P *= 0.005), hypertension (*P *= 0.005) and HCV co-infection (*P *= 0.013). However, there was no difference between the two groups with respect to alcohol use (*P *= 0.105), smoking (*P *= 0.597), BMI (*P *= 0.105), CD4 cell count (*P *= 0.207), and types of ART (*P *= 0.659) (Table 2).

**Table 2:** Prevalence of diabetes among HIV-infected patients on ART at DRH in Northeast Ethiopia

Characteristics	Diabetes, n (%)	No diabetes, n (%)	*P*-value
Age (years)			< 0.001
≤ 45	19 (5.8)	311 (94.2)	
> 45	17 (21.8)	61 (78.2)	
Sex			0.129
Male	16 (11.9)	119 (88.1)	
Female	20 (7.3)	253 (92.7)	
Residence			0.600
Rural	9 (10.2)	79 (89.8)	
Urban	27 (8.4)	293 (91.6)	
Education			0.039
< High school	27 (11.2)	213 (88.8)	
≥ High school	9 (5.4)	159 (94.6)	
Duration of infection (years)			0.035
≤ 5	7 (4.8)	138 (95.2)	
> 5	29 (11.0)	234 (89.0)	
Duration on ART (years)			0.002
≤ 5	11 (4.8)	216 (95.2)	
> 5	25 (13.8)	156 (86.2)	
ART regimen			0.659
AZT + 3TC + EFV	6 (9.1)	60 (90.9)	
TDF + 3TC + NVP	3 (4.2)	68 (95.8)	
AZT + 3TC + NVP	9 (10.7)	75 (89.3)	
TDF + 3TC + EFV	7 (9.2)	69 (90.8)	
Other	11 (9.9)	100 (90.1)	
Smoking			0.597
Yes	2 (12.5)	14 (87.5)	
No	34 (8.7)	358 (91.3)	
Family history diabetes			< 0.001
Yes	12 (28.6)	30 (71.4)	
No	24 (6.6)	342 (93.4)	
Body mass index (kg/m^2^)			0.397
< 25 (Normal/underweight)	34 (8.6)	361 (91.4)	
≥ 25 (Overweight/obesity)	2 (15.4)	11 (84.6)	
Hypertension			0.001
Yes	19 (17.7)	102 (84.3)	
No	17 (5.9)	270 (94.1)	
CD4 count (cell/mm^3^)			0.207
≤ 350	19 (7.5)	236 (92.5)	
> 350	17 (11.1)	136 (88.9)	
HCV co-infection			0.013
Yes	2 (40.0)	3 (60.0)	
No	34 (8.4)	369 (91.6)	

In multivariate logistic analysis, older age (> 45 years; AOR = 3.51, 95% CI 1.52–8.10, *P *= 0.003), longer duration on ART (AOR = 2.67, 95% CI 1.16–6.17, *P *= 0.021), a family history of diabetes (AOR = 6.46, 95% CI 3.36–21.29, *P *< 0.001), and hypertension (AOR = 2.62, 95% CI 1.20–5.72, *P *= 0.016) were independently associated with increased risk of diabetes (Table 3).

**Table 3:** Factors associated with diabetes among HIV-infected patients on ART at DRH in Northeast Ethiopia

Characteristics	Unadjusted OR (95% CI)	*P*-value	Adjusted OR (95% CI)	*P*-value
Age (years)				
> 45	4.56 (2.24–9.27)	< 0.001	3.51 (1.52–8.10)	*0.003*
≤ 45	1.00		1.00	
Education				
< High school	2.24 (10.3–4.89)	0.039	2.11 (0.92–4.86)	0.079
≥ High school	1.00		1.00	
Duration of infection (years)				
> 5	2.44 (1.04–5.73)	0.035	1.86 (0.68–5.07)	0.226
≤ 5	1.00		1.00	
Duration on ART (years)				
> 5	3.15 (1.50–6.59)	0.002	2.67 (1.16–6.17)	*0.021*
≤ 5	1.00		1.00	
Family history diabetes				
Yes	5.70 (2.60–12.52)	< 0.001	6.46 (3.36–21.29)	*< 0.001*
No	1.00		1.00	
Hypertension				
Yes	2.61 (1.31–5.22)	0.001	2.62 (1.20–5.72)	*0.016*
No	1.00		1.00	
HCV co-infection				
Yes	4.74 (1.17–44.81)	0.013	6.49 (0.82–51.09)	0.076
No	1.00		1.00	

### Discussion

In this study, we found a high prevalence of diabetes (8.8%) among patients living with HIV on ART attending our clinic in Northeast Ethiopia. The prevalence estimate of diabetes in this study was comparable to 8.5% prevalence reported from Jimma University Specialized Hospital, Southwest Ethiopia [17] and 8% from Wolayta Sodo hospital, Southern Ethiopia [18], but higher than the 5.1% prevalence reported from University of Gondar Hospital, Northwest Ethiopia [19] and 6.9% from Tikur Anbessa Specialized hospital in Addis Ababa, Ethiopia [20]. Our prevalence estimate of diabetes was also higher than those reported in studies conducted in other countries, including 4.0% from ART clinic of Uganda [21], 4.1% and 6.6% from Malawi [22, 23] and 5.0% from Zambia [24], but lower than the 14.5% prevalence reported from Senegal [25] and 18.0% from Tanzania [26]. The observed difference could be due to variation in the lifestyle and ART regimens or due to variation in the age distribution of the studied subjects.

Consistent with previous studies [14, 17, 25, 27, 28], we found an increased risk of diabetes among older patients with HIV on combination ART. Age as a traditional risk factor plays an important role in the development of diabetes among HIV-infected patients, and with increased survival of infected patients treated with combination ART, a rise in diabetes incidence independent of HIV-related influences may occur with ageing of this population [29, 30]. A family history of diabetes was one of the factors found to be independently associated with increased risk of prevalent diabetes. In the present study, 10.3% of our HIV-infected patients were with a family history of diabetes and they did have a significantly increased risk of diabetes. This was not surprising and is in agreement with previous findings, which demonstrated the higher risk of diabetes among HIV-infected persons who had a family history of diabetes [31, 32].

Our results showed that duration of ART use, but not HIV infection, was associated with an increased risk of diabetes. This was consistent with the results of related studies [17, 25, 33, 34]. Supporting this association, previous follow-up studies have demonstrated induction of insulin resistance and a higher risk of diabetes with the long term use of combination ART [35–37]. Another study has also shown that patients who started ART in early years of the epidemic and had a longer exposure to older ART were at the highest risk of developing diabetes [38]. Targeted screening of diabetes in patients with longer exposure to ART is needed in our setting in order to avoid the associated long-term complications that would otherwise occur.

The present study also found that 29.7% of the study participants had hypertension, which was comparable to other related studies reporting prevalence estimates ranging from 15.9 to 34.4% [17, 18, 22, 23, 25]. The present study also found a significant association between hypertension and prevalent diabetes. This was also supported by other previous studies, which showed that hypertension play a significant role in the development of diabetes among HIV-infected patients on ART [17, 19, 27]. Hypertension screening and treatments are therefore needed to reduce the observed contribution to high diabetes risk in this population.

### Conclusions

In conclusion, our study identified a high prevalence of diabetes (8.8%) among HIV-infected adults attending ART clinic of a hospital in Northeast Ethiopia. Factors such as increased age, family history of diabetes, duration of ART, and hypertension were found to contribute to this high prevalence of diabetes. These findings highlight the need for routine screening and assessment of factors for diabetes in HIV-infected persons receiving combination ART (Additional file 1).

## Limitations

Given the drugs were studied in combinations; we were unable to identify which one contributes to diabetes risk. The other limitation was the cross-sectional nature of the study. The subjective nature of the self-reported response for some items might also be limited by recall bias. Finally, our sampling technique was convenient; therefore, our sample cannot be considered representative of all HIV-infected patients receiving treatment in the country. However, the study has provided some data to inform decision-makers to improve current care and management of HIV-infected persons on ART.

## Additional file

**Additional file 1.** Questioner used in the study.

## Data Availability

The datasets generated and/or analysed during this study are not publicly available due to presence of sensitive (confidential) participants’ information and additional data than that used in this publication. But data are available from the corresponding author on reasonable request.

## Abbreviations

AOR: adjusted odds ratio
ART: antiretroviral therapy
BMI: body mass index
BP: blood pressure
CI: confidence interval
DRH: Dessie Referral Hospital
HCV: hepatitis C virus
HIV: human immunodeficiency virus
