# Supplementary material for: Diabetes mellitus and its associated factors among human immunodeficiency virus-infected patients on anti-retroviral therapy in Northeast Ethiopia
Source: BMC Res Notes. 2019 Jul 1;12:372. doi: 10.1186/s13104-019-4402-1 (PMC6604311; doi:10.1186/s13104-019-4402-1)
Supplement: Supplementary file 1 — Additional file 1. Questioner used in the study. [file 13104_2019_4402_MOESM1_ESM.pdf]

Questionnaire and Format for a Study on Diabetes Mellitus and associated risk factors among HIV infected patients on ART at Dessie referral hospital, Dessie, Northeastern Ethiopia, 2018.

Questionnaire identification Number (ID)____________

Q.	QUESTION	RESPONSE CODE (Check or circle the Appropriate Option)
PART 1- Questions Related to Socio-Demographic Characteristics		
100	What is the sex of the respondent?	1. Male □<br>2. Female □
101	What is the age of the respondent?	Age in years (18-99) ________ (yy.m)<br>(write "99+", if >99 years)
102	Residence of the respondent?	1. Urban □<br>2. Rural □
103	Marital Status of the Respondent	1. Single □<br>2. Married □<br>3. Widowed □<br>4. Divorced □
104	Educational status of the respondent?	1. Illiterate □<br>2. Read & write □<br>3. Grade1-8 □<br>4. Grade 9-12 □<br>5. College & above □
105	Monthly income of the Respondent (in ETB)	1. <1000 □<br>2. 1001-2000 □<br>3. >2000 □
106	Have you ever smoke cigarette in the past 12 month?	1.Yes □<br>2. No □
107	Do you drink alcohol?	1.Yes □<br>2. No □<br>If yes, frequency per week _______
PART 2: Questions related to other risk factors		
200	Duration of HIV infection/disease.	1. < 5 years □<br>2. 5-10 years □<br>3. > 10years □
201	Duration on ART/HAART	1. < 5 years □<br>2. 5-10 years □<br>3. 3. > 10years □
202	ART/HAART regimen (from chart)	1. AZT/3TC/EFV □<br>7. TDF/3TC-ATV/R □

		2. TDF/3TC/NVP 8. ABC/3TC-ATV/R 3. AZT/3TC/NVP 9. ABC/3TC/EFV 4. TDF/3TC/EFV 10. AZT/3TC-ATV/R 5. D4T/3TC/NVP 11. DDI/ABC-ATV/R 6. D4T/3TC/EFV
203	WHO clinical stage (from chart)	1. Stage I 3. Stage III 2. Stage II 4. Stage IV
204	Presence of co infection (from chart)	1. HBV 2. HCV 3. TB
205	Family History of Diabetes Mellitus	1.Yes 2. No
206	Family History of Hypertension	1.Yes 2. No
207	Are you currently taking any kind of blood pressure-lowering medication?	1.Yes 2. No
**PART 3: Clinical and Laboratory Measurements**		
300	Current blood Pressure measurement (Systolic/Diastolic)	1st.________ mmHg 2nd.________ mmHg 3rd.________ mmHg Average_________ mmHg
301	Weight	_____________kg (kilogram)
302	Height	____________m (meter)
303	CD4 cell count	___________cells/mm^3
304	Fasting Plasma Glucose Level	___________mg/dl
